# Successful Rhinophyma Treatment Utilizing the Versajet II Hydrosurgery System: A Case Report and Systematic Review of the Literature

**DOI:** 10.7759/cureus.63921

**Published:** 2024-07-05

**Authors:** Pedro Fuenmayor, Rafael Gottenger, Zoe Pujadas, Bhawna Varma

**Affiliations:** 1 Plastic and Reconstructive Surgery, Larkin Community Hospital, Miami, USA; 2 Plastic Surgery, Baptist Health South Miami Hospital, Miami, USA; 3 Plastic Surgery, Hospital Universitario de Caracas, Caracas, VEN; 4 Plastic Surgery, Larkin Community Hospital, Miami, USA

**Keywords:** rhinophyma, rosacea, versajet ii hydrosurgery system, surgical debulking, tangential excision

## Abstract

Rhinophyma, characterized by hypertrophy of sebaceous glands, often necessitates surgical intervention. This is the second case report of the off-label use of the Versajet II Hydrosurgery System (VJHS) (Smith & Nephew, London, UK) in the United States for the treatment of rhinophyma and the first systematic review of the literature, emphasizing its efficacy and safety for this indication.

A surgical debulking and resurfacing was performed on a patient with rhinophyma. The patient underwent general anesthesia along with bilateral infraorbital blocks and local infiltration of lidocaine 1% with epinephrine. The VJHS was utilized for progressive debulking followed by debridement using sharp instruments until the desired nasal form and contour were achieved. Hemostasis was obtained through monopolar electrocautery and topical hemostatic agents. The patient exhibited excellent nasal shape and healing following VJHS debulking and without perioperative complications, suggesting both the effectiveness and safety of the VJHS in rhinophyma treatment.

A literature review was conducted using the PubMed Central database. Preferred Reporting Items for Systematic Reviews and Meta-Analyses (PRISMA) guidelines, employing inclusion and exclusion criteria, were utilized to narrow down results to include original studies discussing rhinophyma surgical debridement with the VJHS. Six articles were included in the review for results analysis. This case report aligns with findings from international literature, emphasizing the versatility of the VJHS in rhinophyma treatment. Notably, this report marks the second documented off-label use of the VJHS in the United States for rhinophyma.

The success of this case reinforces the potential of the VJHS in treating rhinophyma. This innovative approach yielded promising outcomes in several international reports. Further research is warranted to establish a standardized protocol to validate the long-term benefits of this technology applied to rhinophyma patients.

## Introduction

Rhinophyma is a term formed by the combination of the Greek words "rhis" and "phyma," which mean "nose" and "growth," respectively, and it corresponds to the final stage of acne rosacea [1]. The advanced stage of rosacea can manifest with thickened skin and overactive hyperplastic sebaceous glands that result in an enlarged, bulbous nose primarily in males that frequently requires surgical intervention for correction [2]. Cases of severe rhinophyma nasal deformity can lead to airway obstruction and aesthetic and architectural disfigurement that can create significant psychosocial morbidity and reduced self-esteem [3]. Rhinophyma is more common in White males between the fifth and sixth decade, with a male-to-female ratio varying from 5:1 to 30:1, which is presumed due to increased androgenic hormonal exposure [4].

While recent rhinophyma treatment algorithms include early medical management, definitive treatment is achieved through debulking [1]. Surgical debulking can be either full or partial thickness, followed by application of skin graft or substitutes, or healing by secondary intention [5]. In the United States, a variety of surgical debulking interventions have been used, including dermaplaning, dermabrasion, cryosurgery, electrosurgery, erbium:yttrium-aluminum-garnet (Er:YAG) ablative laser surgery, and sharp debulking [6].

In this paper, we report a successful case of rhinophyma treated with surgical debulking and resurfacing using the Versajet II Hydrosurgery System (VJHS) (Smith & Nephew, London, UK). The VJHS utilizes a high-pressure water jet, created by a Venturi effect on a narrow slit at the tip of the handpiece. When this jet comes into contact with the wound, it allows for the selective tangential excision of tissue in a controlled manner. Simultaneously, the solution is evacuated, minimizing saturation and contamination of the surgical field. The system has been used in the treatment of acute and chronic wounds, burns, wound bed preparation, and general soft tissue debridement [7,8]. To the best of our knowledge, at the time of writing this paper, this is the second case report in the United States and the first systematic review of the literature on the off-label use of the Versajet II Hydrosurgery System (VJHS) for the treatment of deforming rhinophyma.

## Case presentation

**Figure 1:**
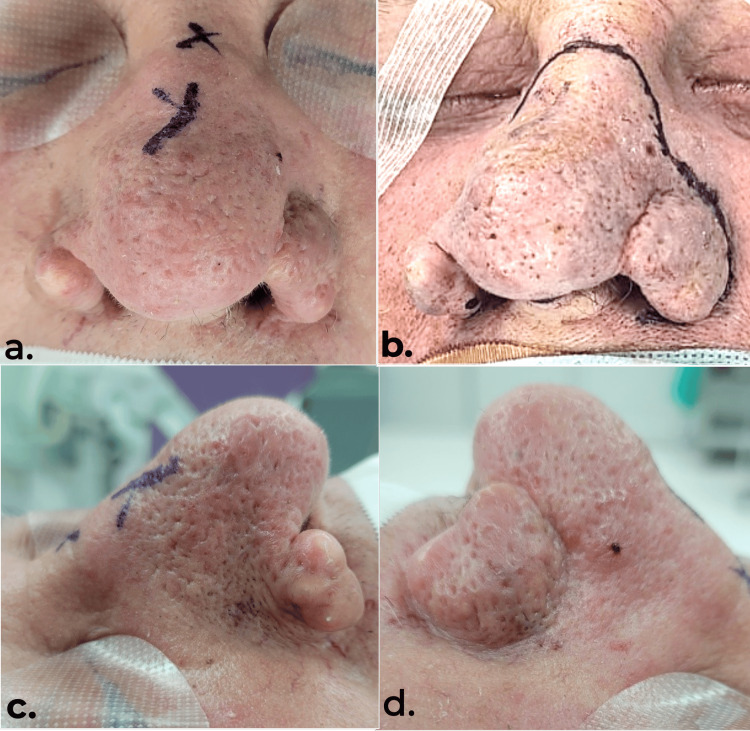
Preoperative views of the rhinophyma in the operating table Frontal (a and b) and lateral (c and d) views showcasing the trophic changes including thickened skin with erythema and telangiectasia, and irregular contours along the tip, dorsum, and alar subunits of the nose.

A pleasant 86-year-old White male with a history of nasal rosacea for more than 50 years presented to our practice with progressive trophic changes toward the nose that have worsened over the last 30 years. The patient found the condition to be significantly noticeable and aesthetically displeasing, prompting them to seek surgical options. On physical examination, the patient presents with an enlarged lower half of the nose, thickened skin with erythema and telangiectasia, and irregular contours along the tip, dorsum, and alar subunits while maintaining patent nares (Figure 1). The patient did not receive any prior medical or surgical treatment. The decision was made to perform a surgical debulking and resurfacing using the VJHS, followed by refinement with sharp surgical instruments.

**Figure 2:**
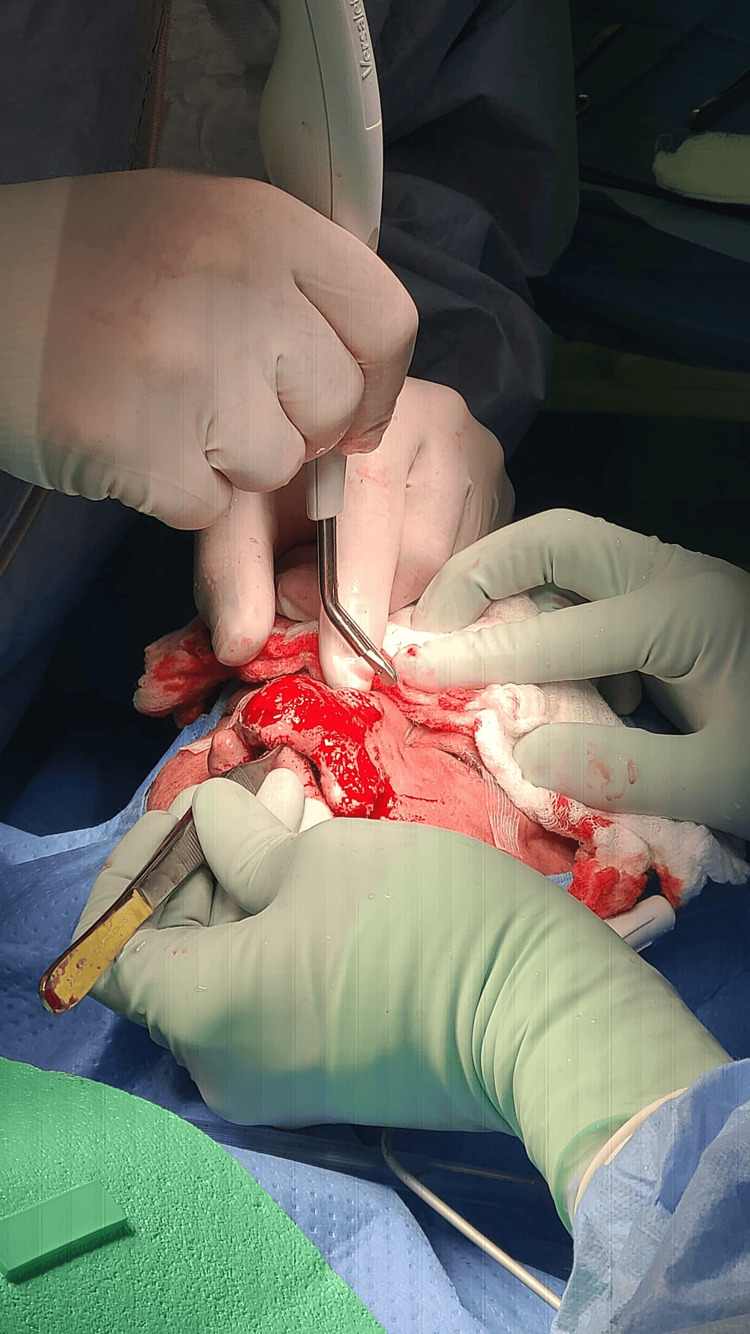
Intraoperative view showing the angulation of the handpiece, highlighting its role in refining the nose contour One of the most ingenious features of the VJHS is the simultaneous suctioning during the water-jet debridement. This prevents oversaturation of the surgical field with blood residues, improving surgical precision and minimizing personnel exposure to blood or aerosolized particles. VJHS: Versajet II Hydrosurgery System

The patient was taken to the operating room and positioned in decubitus, general anesthesia was induced, and the face was prepared and draped in a sterile fashion. Then, bilateral infraorbital nerve blocks and local subcutaneous infiltration of anesthesia were completed utilizing a total of 10 mL of a solution composed of equal parts of lidocaine 1% and bupivacaine 0.5% with epinephrine 1:100,000. After a 10-minute interval, the VJHS with a 45° and 8 mm handpiece was employed for the purpose of debulking the rhinophyma mass, alternating between 1 and 3 of potency depending on the desired depth (Figure 2).

**Figure 3:**
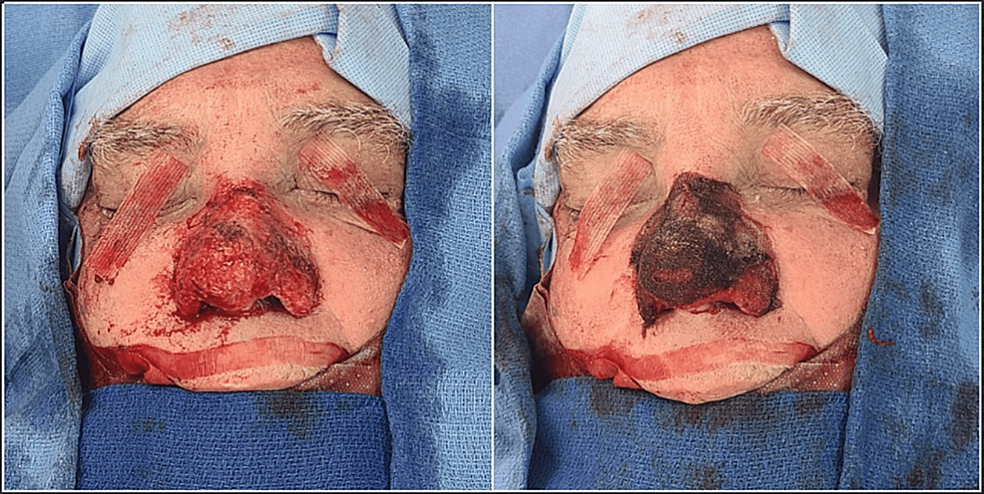
Intraoperative view following complete nasal resurfacing and resulting bleeding in an anatomical area difficult to cover with traditional dressing (left) and the immediate hemostatic effect of topical oxidized regenerated cellulose (Surgicel) and human fibrin sealant glue application (right)

The debulking started at the tip subunit, progressed to the alar subunits, and concluded with the dorsum. Further refinement was complemented with the use of Iris scissors and a #15 knife. Once a satisfactory nasal contour was obtained, our focus shifted to achieving hemostasis employing monopolar electrocautery, followed by the application of topical oxidized regenerated cellulose (Surgicel; Ethicon, Cincinnati, OH) and human fibrin sealant glue (Vistaseal; Ethicon, Cincinnati, OH) as seen in Figure 3.

**Figure 4:**
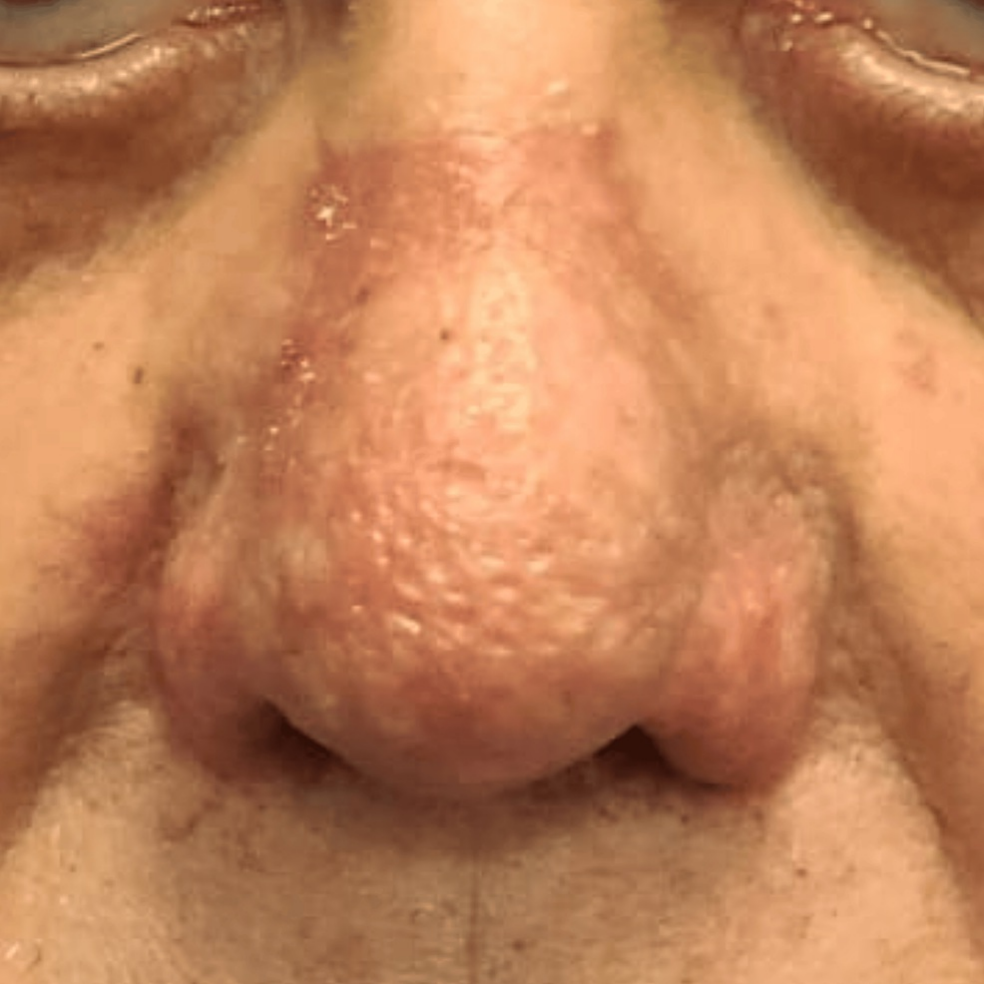
Postoperative view at three weeks displaying smooth nasal contour The patient demonstrated complete healing and high satisfaction with the aesthetic results. Only noticeable is an area of hyperpigmentation that was self-limited and not noticeable after three months.

Then, the surface of the nose was covered with a layer of sterile occlusive gauze with 3% bismuth tribromophenate and petrolatum (Xeroform; Covidien-Medtronic, Dublin, Ireland) and sterile non-adhesive gauze (Telfa; Medline, Northfield, IL). The procedure was well tolerated without any complications. The patient exhibited re-epithelialization within eight days and complete healing by two weeks. During the third-week follow-up visit, the patient expressed a high level of satisfaction with the aesthetic results, with transitory hyperpigmentation of the skin tone that was self-limited as evidenced on later visits (Figure 4). There has been no recurrence of nasal hypertrophy after three months.

## Discussion

Literature review

**Figure 5:**
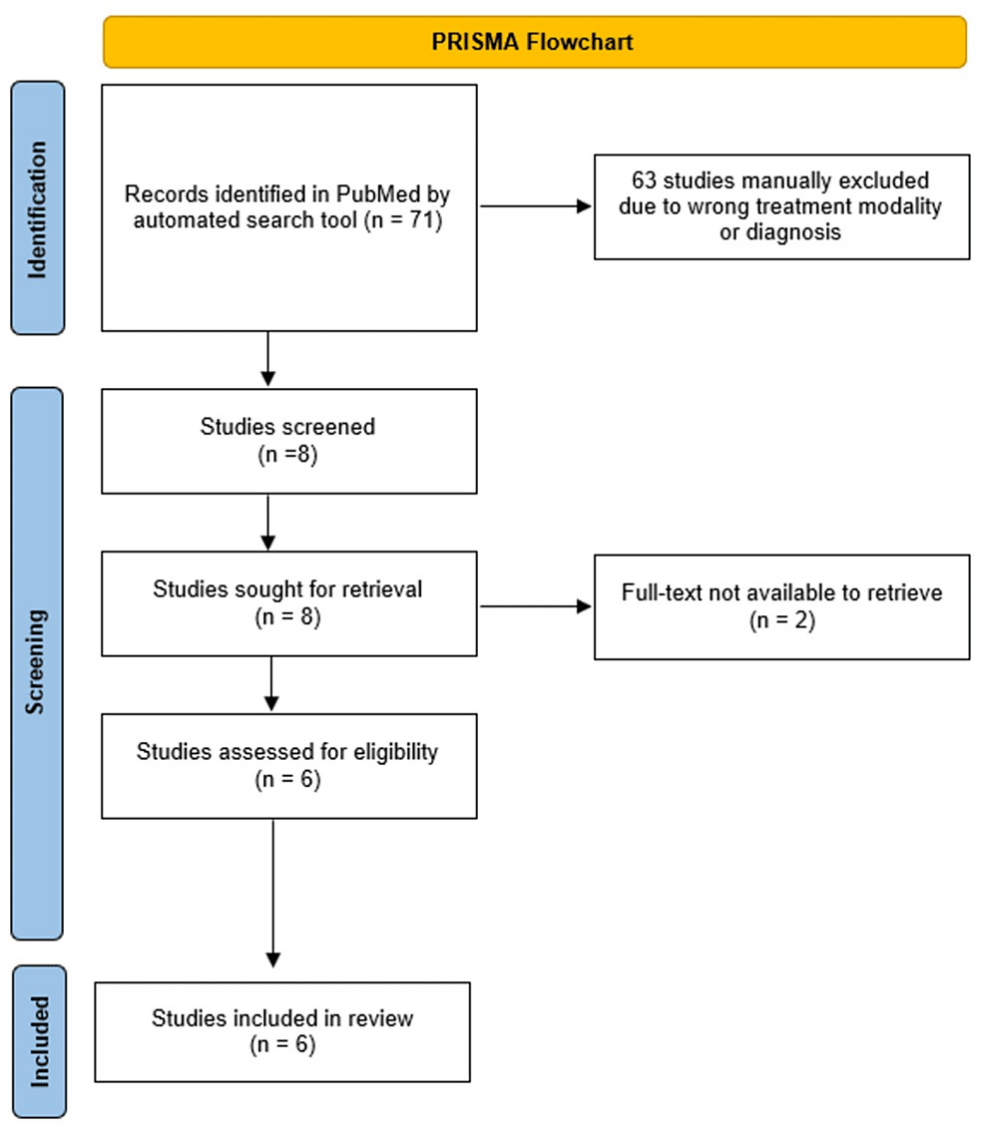
PRISMA flowchart for the systematic review of the literature A systematic review of the literature was performed using the PubMed database, and search terms included "Rhinophyma," "Surgery," OR "Debulking," "Versajet" OR "Hydrosurgery System." PRISMA: Preferred Reporting Items for Systematic Reviews and Meta-Analyses

A comprehensive literature search following Preferred Reporting Items for Systematic Reviews and Meta-Analyses (PRISMA) guidelines using the PubMed database was conducted, employing keywords such as "Rhinophyma," "Surgery," OR "Debulking," "Versajet" OR "Hydrosurgery System" as illustrated in Figure 5. Inclusion criteria comprised articles published in peer-reviewed journals that discussed rhinophyma treatment using VJHS alone or in combination with other modalities. Studies that did not utilize the VJHS were excluded. Data gathered from the literature review was summarized in an evidence table.

Summary of the literature review

A total of 71 studies were identified by automated search tools; of the total, 63 studies were manually excluded due to wrong treatment modality or diagnosis, and eight were assessed for eligibility. Two full-text articles were unavailable, while six articles were retrieved and included for the review. The total patient population comprises 13 patients, of which 12 identified themselves as males and one as female, with ages between 40 and 88 years and an average of 71 years old. Comorbidities among the population including a history of rosacea, cardiac disease, diabetes, and varying degrees of alcohol consumption were reported. Prior to the surgical procedure, five of the 13 patients were prescribed different types of topical and oral treatments including antibiotics, lasers, steroids, retinoids, and vitamins, which were ultimately ineffective in preventing the progression of rhinophyma. The VJHS procedure was performed either under general anesthesia, local infiltrative anesthesia, regional nerve block, or a combination thereof, with good tolerance and no perioperative complications. Wound epithelialization was reported within six and 14 days, and complete healing between three and four weeks following the procedure. Two patients received sprayed processed skin cells (ReCell) as an adjuvant to epithelialization, obtaining full epithelial coverage by day 9 and full healing by the third week [9,10]. All patients in the review reported high levels of satisfaction with the aesthetic results. Table 1 summarizes the reviewed data.

**Table 1:** Evidence summary from the literature review on rhinophyma treated with the Versajet II Hydrosurgery System UK: United Kingdom, N: total number of patients, M: males, F: females, EtOH: ethyl alcohol, VJHS: Versajet II Hydrosurgery System, BCC: basocellular skin cancer, DM: diabetes mellitus, Afib: atrial fibrillation, BPH: benign prostatic hyperplasia

Author	Country	Study design	Population	Comorbidities/social	Anesthesia	Interventions	Outcome/results
Taghizadeh et al. (2008) [6]	UK	Case series	N = 6 (male), age: 41-77	Rosacea, EtOH	General = 3, local = 3, infiltrative = all 6 with 2% lidocaine + 1:80,000 epinephrine + 0.5% bupivacaine	Initial debulking with the scalp for histology sampling. VJHS dermaplaning followed by topical epinephrine for hemostasis. Alginate dressing then weekly dressing change.	5/6 healed by three weeks, one by the fourth week. No complications. No recurrences after a year. All reported excellent aesthetic results.
Dunne et al. (2013) [10]	UK	Case report	N = 1 (male), age: 68	Rosacea, EtOH	Not reported	Oxytetracycline course prior to surgery. VJHS sculpting, followed by topical adrenaline for hemostasis. Adjuvant sprayed processed skin cells (ReCell). Telfa gauze dressing.	Well-formed epithelial buds by the sixth day. Fully healed by the ninth day. Only two dressing clinic visits. Excellent aesthetic result.
Wong et al. (2015) [11]	New Zealand	Case series	N = 3 (1 female/2 male), age: 55-86	Rosacea, BCC, DM, Afib + history of stroke, female with Maori ancestry	General = 2, sedation + local = 1	Topical metronidazole + oral doxycycline for four years previous to surgery. VJHS surgical excision. One case underwent BCC excision, followed by VJHS tangential excision. Daily wound dressing change.	Complete second intention healing by four weeks. No significant scarring. Excellent aesthetic results. No progression after four years following treatment.
Novatti et al. (2015) [12]	Italy	Case report	N = 1 (male), age: 88	Rosacea, EtOH, BPH, heart disease, mechanical nasal obstruction	Infraorbital block	Topical antibiotic ointment before surgery. Electrocautery excision of giant nasal masses obstructing both nares, followed by VJHS nasal remodeling. Dressing with paraffin gauze + gentamicin ointment + intranasal sponges.	Complete epithelialization in two weeks. Improved blood oxygenation after nasal obstruction resolved. Excellent aesthetic result. Improved quality of life.
Yıldız et al. (2017) [9]	Turkey	Case report	N = 1 (male), age: 63	Rosacea, smoking	General	Topical antibiotics, steroids, laser therapy, oral antibiotics, and vitamins before surgery. VJHS dermabrasion, followed by topical epinephrine for hemostasis. Adjuvant sprayed processed skin cells (ReCell). Tie-over dressing.	Dressing changes weekly. Complete healing in three weeks.
Bittar et al. (2020) [13]	USA	Case report	N = 1 (male), age: 75	Rosacea	Not reported	Topical metronidazole, isotretinoin, and oral minocycline before surgery. VJHS tangential excision.	Complete healing by four weeks with significant improvement of nasal, chin, and cheek contour.
Total			13 patients (12 males/1 female)				

This case report aligns with findings from the literature review, emphasizing the versatility and efficacy of the Versajet II Hydrosurgery System in rhinophyma treatment. The existing literature consists only of case reports and small case series, which limits our ability to make definitive conclusions about efficacy, safety, and long-term outcomes. The literature review highlights the ease of use of the system, particularly the adjustability of the hydraulic jetstream power settings. High power is used for tangential excision of thickened skin, while lower power is employed for sculpting and smoothing dorsal and alar contours. Another advantage is the reduced surgical time due to the unobscured view of the highly vascular nasal surgical site, which is made possible by the continuous aspiration of blood and debris by the VJHS. The VJHS has the added benefit of not producing any heat, smoke, or aerosolized blood or blood-borne pathogens, ensuring patient and personnel safety in the operating room. Another advantage is that the intensity of the debridement can be directly controlled by the surgeon using a foot pedal connected to the console or by the operating room nurse on the power unit, allowing for cycling between more or less aggressive excision as needed. Additionally, having the surgical field less saturated with blood allows for more controlled debridement and less tissue loss [14].

In the postoperative period, all patients in the review expressed satisfaction with aesthetic outcomes. The articles that included long-term follow-up data showed none to minimal recurrence or progression of rhinophyma after surgical debulking with the VJHS. All patients included in this review who received medical treatment showed no response to topical or oral medication for preventing progression or reducing nasal hypertrophy; however, all patients showed no recurrence after surgical debulking of the hypertrophied sebaceous gland and thickened skin. The reviewed articles addressed the initial cost of the VJHS console and the recurring expense of the single-use handpiece, which may be offset in institutions already employing the VJHS for burns management. As an additional finding, the two case reports that incorporated sprayed processed skin cells (ReCell) after VJHS experienced a faster healing time. Further studies are warranted to assess the significance of these shorter healing times, and a cost analysis should be conducted to justify its standard use.

## Conclusions

The success of this case reinforces the potential of the Versajet Hydrosurgery System in treating rhinophyma. Notably, this report marks the second documented off-label use of Versajet in the United States for rhinophyma, contributing to the limited body of literature. This surgical approach yielded excellent outcomes both in the presented case and in the reviewed literature. The Versajet Hydrosurgery System allowed for controlled debridement and nasal sculpting, resulting in aesthetically pleasing outcomes. This system is convenient, practical, and easy to use, providing the surgeon with significant control over the excision. This ultimately optimizes the efficacy and safety of the procedure. These findings underscore the potential benefits of adopting such technology in the surgical treatment of rhinophyma, as evidenced by the consistently positive results reported across multiple cases. Further research is warranted to establish standardized protocols and validate the long-term benefits of this off-label use. Additionally, future studies should aim to compare different treatment modalities to provide a more comprehensive understanding of the most effective and cost-efficient treatment options for rhinophyma.
